# Clinical and demographic factors associated with sexual behaviour in children with autism spectrum disorders

**DOI:** 10.4102/sajpsychiatry.v23.862

**Published:** 2017-02-06

**Authors:** Lurike Fourie, Carla Kotzé, Deborah van der Westhuizen

**Affiliations:** 1 Department of Psychiatry, School of Medicine, University of Pretoria, South Africa

## Abstract

**Introduction:**

The sexual behaviour and development of children with autism spectrum disorders (ASDs) have been mostly overlooked in research and practice. This study aimed to determine the association between certain clinical and demographic factors found in a sample of children with ASDs, and their reported sexual behaviour (RSB).

**Methods:**

The study was conducted at a school in Gauteng, South Africa, for learners with ASDs. Two questionnaires completed by caregivers/parents enquired about family stability, clinical profile and RSB (if any) in their child. RSB was analysed via three domains: self-care, socio-sexual skills and actual RSB, with additional information from school records.

**Results:**

Of the 107 questionnaires distributed, only 31 parents responded and 24 agreed to participate. The 24 (100%) children included 10 pubertal and 14 pre-pubertal children, of which 18 (*n* = 18) had more stable primary caregiver statuses as well as more stable socioeconomic and family environments. Two of the 14 pre-pubertal children had abnormal self-care, whereas none of the 10 pubertal children had any abnormal self-care. Eight of the 18 children from more stable environments displayed abnormal sexual behaviours. Of the 6 children from less stable environments, two displayed more abnormal socio-sexual skills, whereas 9 of the 18 children from more stable environments displayed more abnormal sexual behaviour. In contrast with the postulated hypothesis that children from less stable socioeconomic and family environments would exhibit more abnormal sexual behaviours, this study did not find any evidence of such a relationship or association.

**Conclusion:**

ASDs are characterised by deficits in communication and social skills. These may lead to an affected individual struggling to develop appropriate sexual behaviour. If specific risk factors that contribute to the development of abnormal sexual behaviour can be identified, one can try to modify/prevent these where some degree of prevention or alleviation may be possible.

## Introduction

In the past, it was assumed that children/adolescents with Autism Spectrum Disorders (ASDs) showed little interest in sexuality.^1^

Children with ASDs all go through the same process of sexual maturation and development as neurotypical adolescents.^2,3^ A survey done by Haracopos and Pederson^4^ showed that adolescents with ASDs have the desire to have intimate relationships with others and are interested in sexual behaviour. This has been confirmed by other studies and current research indicates a high rate of sexual behaviour among this population.^1,3,4,5,6,7,8^

Social awareness, reciprocal social interaction and adequate communication skills are essential in the learning and understanding of appropriate sexual behaviour. Unfortunately, these are exactly the areas in which individuals with ASDs show impairment. This leads to the individual struggling to achieve a social and sexual identity.^3,9,10,11^ Many may then behave in a way that is deemed to be sexually inappropriate. The inappropriate sexual behaviours, as well as the caregivers’ lack of knowledge of the sexual development and behaviour of their children with ASDs are of significant concern.^1,2^

The problem in defining ‘inappropriate sexual behaviour’ is firstly to identify what can be regarded as ‘appropriate’ sexual behaviour. This may be likely to differ between different cultural, religious and social groups. Inappropriate sexual behaviour may not have a sexual basis but may serve a communicative function or may reflect a general lack of knowledge of appropriate behaviour.^12^

Sexual inappropriateness and hypersexuality are defined by The California Evidence-Based Clearinghouse for Child Welfare^13^ as vigorous sexual drive or other sexually related problems that interfere with normal activities of daily living, or sexual behaviour that is pursued at inappropriate times. By definition, such behaviour is persistent, uninhibited and directed against oneself or towards unwilling partners. Inappropriate sexual behaviour encompasses a range of behaviour, including suggestive language, flirtation, fondling, removing one’s clothing and masturbating in public.^14^

It should be noted that although the term ‘sexual’ is used, the reasons for engaging in such behaviour vary and are rarely related to sexual gratification or stimulation. Instead, these behaviours tend to be related to other factors, such as curiosity, impulsivity, anxiety, trauma-related symptoms (e.g. re-experiencing symptoms of post-traumatic stress disorder) and attention-seeking.^14^

Few studies have been done internationally, and even fewer in South Africa on the topic of sexual development and behaviour in ASDs. The sexual drives that emerge in individuals with ASDs are not accompanied by the socialisation and understanding of social norms that usually govern what are deemed acceptable sexual behaviours.^1^ Further research may improve our understanding of the issues surrounding the sexual development and behaviour of children with ASDs, as well as decrease the stigma thereof and the reluctance of caregivers to discuss the sexual behaviour of their children.

## Methods

This quantitative study was based on questionnaires completed by caregivers, as well as data collected retrospectively by the researcher from school records. The study was performed at a school, which caters for about 100 learners, that offers specialised education to learners with ASDs aged between 3 and 18 years. The research protocol was approved by the Ethics Committee of the Faculty of Health Sciences of the University of Pretoria. Permission was obtained from the school principal and parents/caregivers to access records of students. All children attending the school with ASDs, whose parents/caregivers gave informed consent, were included in this study.

Two questionnaires were completed by the caregivers/parents of each child. Caregiver, for the purposes of this study will refer to the mother or grandmother as identified via the completed questionnaires received. The first questionnaire obtained demographic as well as clinical data about each research participant. The demographic data included age; socioeconomic circumstances/status of family; and marital, educational, financial and employment status of parents/primary caregivers. The clinical data that were collected by the researcher included the child’s psychiatric diagnosis; comorbid psychiatric illness; current medication; and family psychiatric history. The presence of sexual abuse, exposure to domestic violence and neglect was also explored.

The second questionnaire comprised questions used in the Child Sexual Behaviour Inventory (CSBI),^15^ an inventory that is used in children where sexual abuse is suspected, as well as the Interview of Sexuality in Autism Revised (ISA-R).^16^ In this questionnaire, the three domains that were assessed included the child’s self-care; socio-sexual skills; and lastly the RSB of each child. An enquiry was also made into the presence of any physical changes related to puberty and, if present, the child’s response to these changes.

The following parameters were used to analyse the socioeconomic circumstances and the environment of the families: availability of basic sanitation, type of dwelling, number of rooms in the dwelling, number of people living in the dwelling, source of income, history of sexual abuse, history of domestic violence and history of neglect. As there are eight questions, a score was given out of eight to determine whether the socioeconomic and family environments were stable or unstable (less than 8 = less stable; 8 = more stable) (Table 1).

**TABLE 1:** Socioeconomic circumstances of family, neglect/abuse.

Factor highlighted	Equal to 8 items = more stable	Less than 8 items = less stable
Basic sanitation (water, electricity, refuse removal)	Yes	Only certain services; no service available
Type of dwelling	RDP (Reconstruction and Development) house, brick house, flat/apartment	Shack
Amount of rooms in dwelling	2 or more than 2 rooms	Less than 2 rooms
Number of people living in the dwelling	4 and less	More than 4
Source of income of parent/caregiver	Salary	State grant/pension Donations/charity
History of sexual abuse	No	Yes
History of domestic violence	No	Yes
History of neglect	No	Yes

The information from the questionnaires was combined with additional/ relevant information from the school’s staff records, for example, old school reports and learner progress reports.

Children with a clinical diagnosis other than ASDs (e.g. schizophrenia) and children whose parents/caregivers were unavailable or not able/willing to give informed consent were excluded.

## Results

Of the 107 questionnaires handed out, only 31 parents responded and 24 agreed to participate in the study.

The aim of the study was to determine if there is any association between certain demographic and clinical factors found in a specific population of children with ASDs, and their sexual behaviour.

### Demographic data

The demographic profile of each child’s family (with the main focus on the socioeconomic circumstances of the family), as well as demographic status of the primary caregiver were drawn up.

The study attempted to explore the demographic status of the primary and secondary caregivers of each child. The parameters included relation to the child, employment status and educational level. Not enough information was available to draw reliable conclusions for the secondary caregiver and the focus was therefore only on the primary caregiver. As three parameters were explored, less than 3 was regarded as less stable; equal to 3 was regarded as more stable) (Table 2).

**TABLE 2:** Information about the primary caregiver of the child.

Information about the primary caregiver of the child	3 = more stable demographic status	< 3 = less stable demographic status
Who is the caregiver?	1–5 1. Biological mother 2. Biological father 3. Stepmother 4. Stepfather 5. Grandparent	6–10 6. Another family member (like an aunt/uncle/cousin) 7. A sibling 8. A friend 9. Children’s home staff member 10. Place of safety personnel member
Employment status	Employed	Unemployed, unknown
Level of education	Matric, tertiary	Std. 5; std. 8; less than 12 years; unschooled, unknown

Of the 24 children who were included in the study, the following were noted regarding the primary caregiver:

- Twenty-three children had their mother as the primary caregiver; one child had the grandmother as a primary caregiver.
- Eighteen children had primary caregivers with a more stable demographic status.
- Four children had a primary caregiver with an education level of less than 12 years.
- One child had a primary caregiver who had unstable employment and low educational status.
- One child had a primary caregiver with unstable employment.
- The majority of children thus had a more stable primary caregiver demographic status.

### Self-care, socio-sexual skills and actual sexual behaviour observed

The children were divided into two age groups: Pre-pubertal (3–12 years) and Pubertal (>12–18 years). The reason for this being that certain sexual behaviours may be considered appropriate in pre-pubertal children, whereas the same behaviour may be very inappropriate in pubertal children. Of the 24 children, there were 14 pre-pubertal children.

### The three aforementioned domains were enquired into and were defined as follows

*Self-care* was defined as: ‘any intentional action taken by an individual for that individual’s physical, mental and emotional health’. Self-care is any necessary human regulatory function which is under individual control. It is also deliberate and self-initiated, for example, appropriate use of toilets, the wearing of appropriate clothes and washing of the genital areas. Four questions were asked regarding this.

*Socio-sexual skills* refer to basic communicative and relationship skills such as the ability to read facial expressions and to anticipate emotional responses. These social and interpersonal skills are necessary to integrate into a community successfully. Enquiry was made into nine behaviours in this domain.

*Sexual behaviour*, as mentioned by Hayward Saunders,^10^ included self-image, emotions, values, attitudes, beliefs, behaviours and relationships. From the perspective of parents/caregivers, it would largely be the behaviour they observe in their child(ren). Enquiry was made into 27 behaviours in this domain.

In order to perform statistical tests for the association between demographic factors (socioeconomic and family environment) and the level of abnormal sexual behaviour, the children were divided into two groups:

#### *Less* stable and *more* stable socioeconomic and family environments

Those with *less than eight stable* socioeconomic and family items: less stable. Those with *eight stable* items: more stable.

#### *Less* and *more* abnormal sexual behaviours

Those with *less than four abnormal* sexual behaviours: less abnormal. Those with *four or more abnormal* behaviours: more abnormal.

Only 6 of the 24 children come from *less stable* socioeconomic and family environments and of these six children, only two (33%) exhibited *more abnormal* sexual behaviours.

Nine (50%) of the 18 children from *more stable* environments exhibited *more abnormal* sexual behaviours.

In terms of the three domains enquired into specifically, the results are as follows.

#### Self-care

Of the 24 children, only two (33%) displayed more *abnormal* self-care items. Only 2 of the 14 pre-pubertal children had *abnormal* self-care behaviour and none of the 10 pubertal children had any abnormal self-care.

#### Socio-sexual skills

Eight of the 18 children from *more stable* environments displayed *more abnormal* socio-sexual skills. Of the six children from *less stable* environments, two displayed *more abnormal* socio-sexual skills. Items identified as problematic are summarised in Table 3.

**TABLE 3:** Problem item identified regarding socio-sexual skills.

Number	Problem item identified	Number of children involved	Pre-pubertal	Pubertal
1.	Doesn’t know where it is allowed to be naked	2	1	1
2.	Undresses in public	7	4	3
3.	Sits with crotch/underwear exposed	5	3	2
4.	Undresses others	2	0	2
5.	Touches genitals in the presence of others	7	2	2
6.	Doesn’t know that no one should touch his/her genital areas inappropriately	13	6	2
7.	Makes sexual sounds	1	1	0

*Source*: Adapted from Friedrich et al. (2001)

#### Sexual behaviour

Nine of the 18 (thus 50%) children from *more stable* environments displayed *more abnormal* sexual behaviour. Items identified as problematic are summarised in Table 4.

**TABLE 4:** Problem item identified regarding reported sexual behaviour.

Number	Problem item identified	Number of children involved	Pre-pubertal	Pubertal
1.	Masturbates/self-stimulates	5	1	4
2.	Grabs females breasts	7	3	4
3.	Touches genitals of others	1	0	1
4.	Kisses/hugs strangers	6	4	2
5.	Kisses others with tongue in other person’s mouth	2	1	1
6.	Wants to watch TV programmes with nudity	3	2	1
7.	Wants to look at pictures of nude people in magazines	2	1	1
8.	Asks others to engage in sexual acts	1	0	1
9.	Imitates acts of sexual intercourse	2	1	1
10.	Overly friendly with strange men	1	1	0
11.	Scratches own anal area/crotch area	11	3	8
12.	Spies on others getting undressed	1	0	1
13.	Plays ‘doctor–doctor’ and touches inappropriately	1	0	1
14.	Walks around without clothes on	8	3	5
15.	Intrudes into the ‘personal space’ of others, making them uncomfortable	4	1	3

*Source*: Adapted from Friedrich et al. (2001)

Grabbing of female breasts, kissing/hugging of strangers, scratching of the crotch area or walking around without clothes in the house were deemed as *less abnormal* behaviour in pre-pubescent children.

Those children who displayed four or more items regarded as being more abnormal sexual behaviour are discussed below.

**Child number 1:** One of the children involved was a pubertal female. She started menstruating at the age of 10 years and 11 months. This apparently upset her greatly. She would sometimes scratch her genital area until it bled. This behaviour still continues. She comes from a more stable demographic and family environment.

Her *self-care* at the time of completion of the questionnaire was normal. With regards to *socio-sexual skills*, she struggled to know where she was allowed to be naked or not; she undressed in public at times. She would also touch her genital areas in the presence of others.

With regards to *sexual behaviour*, she would hug/kiss strangers and scratch her genital area. She had to be told where it would be appropriate to dress and undress. Intervention via ‘girl-talk’ with a female psychologist helped to make her aware of her own sexuality and to accept it.

**Child number 2:** This was a pubertal child, currently in a safe, more stable socioeconomic and family environment. The questionnaire completed volunteered the following information:

The child was exposed to domestic violence a few years earlier. The parents had marital problems which at times resulted in physical aggression between them. It would seem that there were significant financial stressors at the time. In this case, social services were not involved, but the teachers working with the child did address issues concerning abnormal or inappropriate sexual behaviour. Psychological intervention is ongoing for this child.

This child displays normal self-care. On socio-sexual skills items, the child would reportedly sometimes sit with underwear exposed, undressed in public once and would sometimes unbutton others’ shirts.

Regarding other observed sexual behaviour: the child would sometimes masturbate or stimulate himself, would grab women’s breasts or scratch his genital area. He once walked naked in the house and sometimes invades others’ personal space making them uncomfortable. Further feedback was that the child was really unsure of himself and lacked knowledge of his own body.

**Child number 3:** This child is a pubertal child from a single parent household (absent father). When the mother is not at home, the child is looked after by a child carer. This child displays the following inappropriate sexual behaviours: looking at pictures of nude people in magazines, imitating the act of sexual intercourse, scratching genital area and sometimes walking in the house without clothes on.

**Child number 4:** This was a male pubertal child, from a more stable demographic and family environment. He displays poor socio-sexual skills: undresses in public, sits with underwear exposed and touches his genital area in the presence of others.

The child also displays abnormal or inappropriate sexual behaviour. He masturbates, sometimes grabs women’s breasts, has put his tongue in another person’s mouth and walks in the house with no clothes on.

**Child number 5:** This was a pre-pubertal child from a more stable demographic and family environment. The child displayed poor socio-sexual skills: sits with underwear exposed, touches genital area in the presence of others and makes certain sounds that can be deemed as ‘sexual’ in nature. Inappropriate sexual behaviour observed: scratching genital area and walking in house with no clothes on.

**Child number 6:** This was a pubertal child from a more stable demographic and family environment. This child displayed a significant amount of inappropriate behaviour. The reported behaviours included: sitting with genital area exposed, undressing others in public and touching own genital area in the presence of others.

Observed sexual behaviour included: grabbing women’s breasts, wanting to watch TV programmes with nudity and looking at pictures of nude people, as well as imitating acts of sexual intercourse.

There were also three separate incidents where the child spied on others who were getting undressed, played ‘doctor-doctor’ with inappropriate touching of another person’s genital area and walked in the house with no clothes on.

It was confirmed that this child has been included in groups run by psychologists at the school to educate the children as to what behaviour is appropriate and in which circumstances or places.

Additional questions in the parent/caregiver questionnaire asked whether or not the child (if pubertal) had been having any physical changes related to puberty and also what the child’s response to these changes have been. The following were some of the replies given:

A male that recently entered puberty had physical changes, for example, hair growth in axillae and deepening of voice. The child displayed some irritable behaviour at times. He would sometimes urinate in inappropriate places and seemed very unsure of himself.

A few other pubescent children displayed a poor sense of self and would try to hide physical changes, for example, development of breasts.

One case of a pre-pubertal child will be highlighted here. In this case, the history of domestic violence and neglect was not indicated in the filled-out questionnaire, but perusal of the school records did show such a history. Social services became involved. It is uncertain at what age the child had been removed from the mother’s care, but the child was taken to alternative care. This child displayed aggressive and oppositional behaviour for several years. The child needed assistance with self-care and displayed sexually inappropriate behaviour (which was not elaborated on).

The child’s environment is now safe and can be regarded as a more stable environment. The questionnaire completed and returned for this study showed the following results: The child displayed normal self-care, but the child previously struggled to know where it is permissible to be naked or not. Regarding sexual behaviour observed: the child once asked to watch programmes with nudity or look at pictures of nude people in magazines. The child would also sometimes imitate an act of sexual intercourse.

For statistical testing, Fisher’s exact test on cross tabulations was performed. The *p*-value of 0.65 does not provide evidence of a statistically significant association/relationship at the 5% level, between stability of socioeconomic and family environment and inappropriate sexual behaviour.

Specific clinical factors were also looked at to determine if there could be a link between sexually inappropriate behaviours (Table 5).

**TABLE 5:** Clinical factors.

Variable	Factors
Autism spectrum disorder	Autism: 21 Asperger’s: 1 PDD NOS (Pervasive Developmental Disorder Not Otherwise Specified): 2
Comorbid psychiatric diagnosis in child	Mood disorders MDD: 1 Obsessive compulsive disorders OCD: 1 Intellectual disability MR: 1 Attention deficit/hyperactivity disorder ADHD: 5
History of family psychiatric illness	Yes: 7 No: 17 Conditions: Mood disorders MDD: 2 BMD: 3 ADHD: 1 Psychotic disorders: 1 (unspecified)

As shown in Table 4, only 5 children had a comorbid diagnosis of ADHD (Attention Deficit Hyperactivity Disorder), whereas 13 children were on stimulant medication.

Only one child had comorbid intellectual disability. Children with ASDs as well as those with intellectual disability may show sexually inappropriate behaviour. A dual diagnosis may be a confounding factor when studying sexual behaviour.^16^ In this study, this was not found to be the case.

Of the 24 participants, only 7 had a history of family member(s) with psychiatric illness. No association was found between the presence or absence of ASDs and family history of psychiatric illness.

## Discussion

This study enquired if an association exists between certain demographic factors (socioeconomic circumstances, caregiver profiles) and levels of abnormal sexual behaviour in children with ASDs.

An expected finding of the study might have been that children from less stable socioeconomic and family environments exhibit more abnormal sexual behaviours. However, this study did not find any evidence of such an association.

An overview of frequently found traits with children/youth who exhibit inappropriate sexual behaviour (ISB) is given by O’Brien (2010). These traits include ‘adverse parental/caregiver circumstances, poor parental/caregiver mental health, unstable living arrangements … grief and loss, recent victimisation’.^17^ Further correlations between children and ISB included family dysfunction and poverty.

As mentioned by Friedrich et al.,^15^ sexual behaviour is significantly related to sexual abuse in children. Any exposure may contribute to a child engaging in sexually inappropriate behaviour. Sullivan et al.^1^ state that children with ASDs are at high risk of being sexually victimised. As many as 16% – 25% of persons with autism have been sexually abused.^1,18^ None of the children in this study were exposed to sexual abuse.

There are several possible explanations as to why sexual behaviours of concern may occur: sexual behaviour may be the only source of pleasure, excitement or gratification available to the person; it may serve to reduce ‘anxiety’; it can allow the young person to feel security in routine activities; inappropriate sexual conduct may be the only alternative available to seeking relationships; a young person may attempt to copy an observed adult sexual behaviour; she or he may attempt to make connections with peers using sexual information and behaviours; she or he may have experienced sexual abuse.^10,12^

## Limitations

A serious limitation of this study was that it was an observational study conducted at one school that caters for children with ASDs. This resulted in a very small sample of respondents; therefore, the results of this study cannot be generalised to a larger population.

One of the demographic factors the authors wanted to compare was that of gender. Unfortunately, only 3 of the 24 children included in the study were female and the rest were all male. Owing to such a skewed representation of females, no gender comparisons could be done.

The sampling was also influenced by the willingness of parents/caregivers to respond. The small sample of respondents in this study may be an indicator as to how reluctant the parents/caregivers of children with ASDs are of talking about/disclosing information on the sexual behaviour of their children. Sullivan and Caterino^1^ stated that sexuality and sexual education are highly emotional and moralistic matters for most. As mentioned by Tissot,^5^ there are other factors that must be taken into account. These include ‘…the sensitive aspects of ISB, as well as the abstract nature of teaching the topic of sexuality in individuals with disabilities’.

Another limitation was that the estimated intelligence quotient scores of the children were not available to the researcher. Intellectual disability may contribute to sexually inappropriate behaviour.^16^

## Conclusion

ASDs are characterised by deficits in communication and social skills. These deficits may lead to the individual struggling to find a sexual identity, as well as impaired development of appropriate peer-group relationships. Abnormal sexual behaviour may then occur.

This study did not find any association between certain demographic and clinical factors in a specific population of children with ASDs, and their sexual behaviour. Future research may, however, yield different results.

If specific risk factors that contribute to the development of abnormal sexual behaviour can be identified, one can try to modify/prevent these where some degree of prevention or alleviation may be possible.
